# A potential three-gene-based diagnostic signature for idiopathic pulmonary fibrosis

**DOI:** 10.3389/fgene.2022.985217

**Published:** 2023-01-06

**Authors:** Yi Wu, Lin Zhong, Li Qiu, Liqun Dong, Lin Yang, Lina Chen

**Affiliations:** ^1^ Division of Pediatric Pulmonology and Immunology, West China Second University Hospital, Sichuan University, Chengdu, China; ^2^ Key Laboratory of Birth Defects and Related Diseases of Women and Children (Sichuan University), Ministry of Education, Chengdu, China; ^3^ NHC Key Laboratory of Chronobiology (Sichuan University), Chengdu, China

**Keywords:** idiopathic pulmonary fibrosis, diagnostic biomarkers, WGCNA, bioinformatics analysis, differentially expressed genes

## Abstract

**Background:** Idiopathic pulmonary fibrosis (IPF) is a life-threatening disease whose etiology remains unknown. This study aims to explore diagnostic biomarkers and pathways involved in IPF using bioinformatics analysis.

**Methods:** IPF-related gene expression datasets were retrieved and downloaded from the NCBI Gene Expression Omnibus database. Differentially expressed genes (DEGs) were screened, and weighted correlation network analysis (WGCNA) was performed to identify key module and genes. Functional enrichment analysis was performed on genes in the clinically significant module. Then least absolute shrinkage and selection operator (LASSO) logistic regression and support vector machine-recursive feature elimination (SVM-RFE) algorithms were run to screen candidate biomarkers. The expression and diagnostic value of the biomarkers in IPF were further validated in external test datasets (GSE110147).

**Results:** 292 samples and 1,163 DEGs were screened to construct WGCNA. In WGCNA, the blue module was identified as the key module, and 59 genes in this module correlated highly with IPF. Functional enrichment analysis of blue module genes revealed the importance of extracellular matrix-associated pathways in IPF. IL13RA2, CDH3, and COMP were identified as diagnostic markers of IPF *via* LASSO and SVM-RFE. These genes showed good diagnostic value for IPF and were significantly upregulated in IPF.

**Conclusion:** This study indicates that IL13RA2, CDH3, and COMP could serve as diagnostic signature for IPF and might offer new insights in the underlying diagnosis of IPF.

## Introduction

Idiopathic pulmonary fibrosis (IPF) is a chronic, progressive, fibrotic lung disease of unknown cause (Spagnolo et al., 2021). The global annual incidence of IPF is estimated to range from 0.2 to 93.7 cases per 100,000 people (Hutchinson et al., 2015), and the median survival time from diagnosis is 4.5 years (Kaunisto et al., 2019). Substantial progress has been made in the clinical management of IPF. Two effective agents, nintedanib and pirfenidone, benefit physiological deterioration and progression-free survival (Noble et al., 2016; Raghu et al., 2017). Timely diagnosis may improve outcomes for patients in the future, but the coexistence of other disorders and the lack of specific symptoms delay diagnosis, which has worsen mortality (Lamas et al., 2011). Current efforts are directed at identifying IPF early, potentially relying on combinations of biomarkers (Martinez et al., 2017), and identifying key biomarkers that may direct more personalized medicine to improve long-term prognosis (Barratt et al., 2018).

Major advances recognizing biological mechanisms and biomarkers have occurred over the past decade with the development of transcriptome analysis (Casamassimi et al., 2017; Raza et al., 2022). Numerous studies have focused on circulating molecular markers or lung-specific sampling to improve the diagnosis of IPF (Ley et al., 2014). These biomarkers include extracellular matrix (ECM)-modifying enzymes (Kropski et al., 2015), matrix metalloproteinase (MMP)-degraded proteins (Jenkins et al., 2015), inflammatory proteins (Li et al., 2022), and the transcriptomic signature (Bauer et al., 2015). However, knowledge of molecular biomarkers for IPF remains in its infancy (Ley et al., 2014).

In this study, we used multiple bioinformatics methods to identify key gene co-expression modules, functional pathways, and significant diagnostic biomarkers in IPF, and constructed a potential diagnostic signature for IPF.

## Materials and methods

### Acquisition of microarray data

**FIGURE 1:**
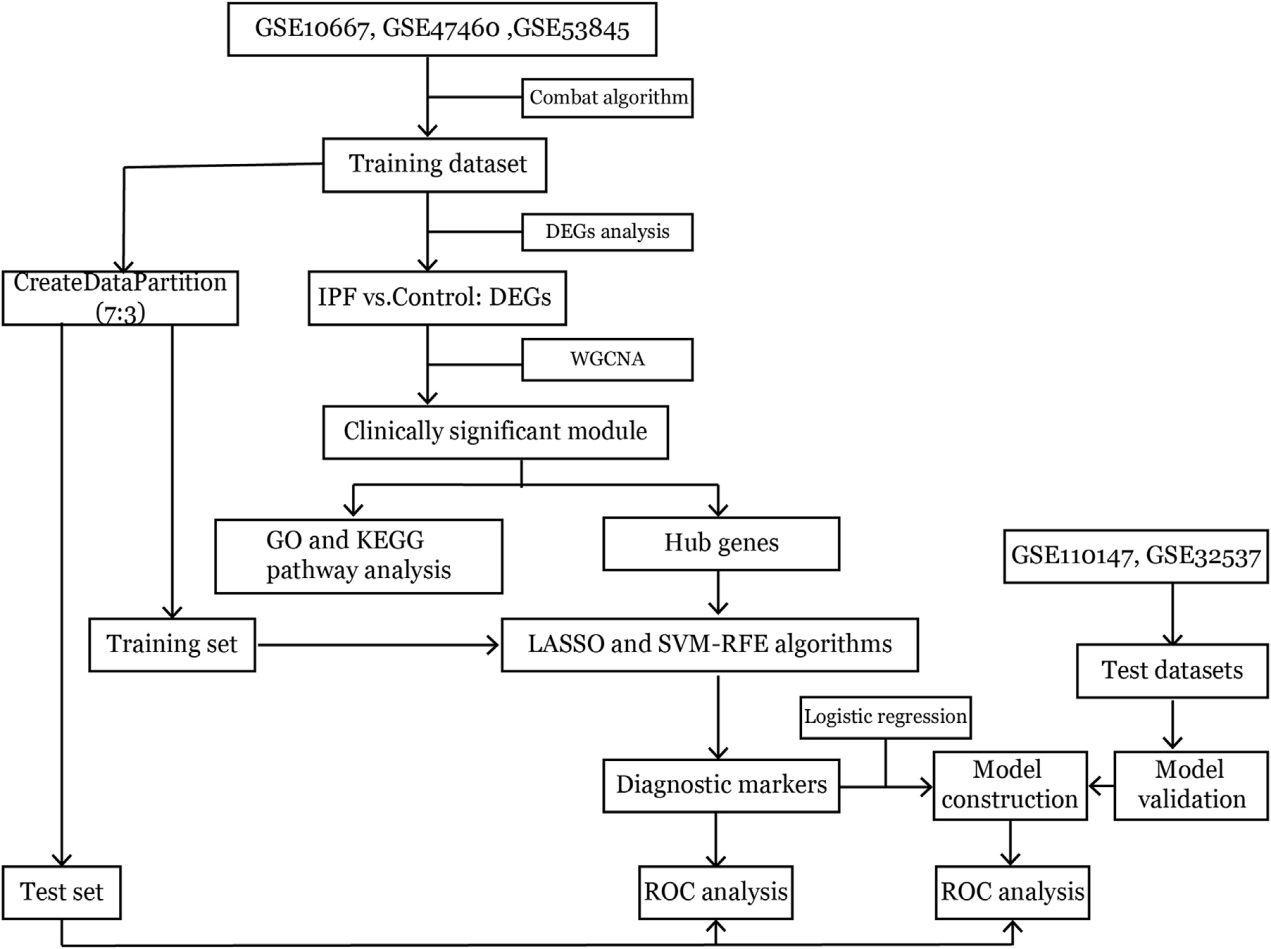
The workflow of analysis.

The workflow of this study is shown in Figure 1. Microarray datasets of IPF-related gene expression profiles were retrieved and downloaded from the NCBI Gene Expression Omnibus public database (https://www.ncbi.nlm.nih.gov/geo/). The included datasets met several selection criteria: 1) the datasets published in the past 15 years; 2) samples had been extracted from lung tissue; 3) the total sample size in two groups was at least 30; and 4) raw data were available in the datasets. Five datasets that met the selection criteria and the diagnostic criteria for IPF were analyzed in this study (American Thoracic Society, 2000; Holmes et al., 2006; Raghu et al., 2011), and information about them was presented in Table 1. Ethical approval for this study was not required because these involved datasets were freely available in public datasets.

**TABLE 1:** Microarray datasets included in this study.

Geo accession	Publication year	Sample size (IPF/Control)	Source	Platform
GSE110147	2018	22/11	Lung tissue	Affymetrix Human Gene 1.0 ST Array
GSE53845	2014	40/8	Lung tissue	Agilent-014850 Whole Human Genome Microarray 4 × 44K G4112F
GSE47460	2013	122/91	Lung tissue	Agilent-028004 SurePrint G3 Human GE 8 × 60K Microarray
GSE32537	2013	119/50	Lung tissue	Affymetrix Human Gene 1.0 ST Array
GSE10667	2009	31/15	Lung tissue	Agilent-014850 Whole Human Genome Microarray 4 × 44K G4112F

### Data preprocessing and differential expression analysis

Raw data from the GSE10667, and datasets were normalized with the normexp function and the between-array-normalization quantile in Limma R package (Ritchie et al., 2015) and merged into a training dataset. These three datasets were selected for integrated analysis because they had the same platform, which is crucial when combining different datasets. Then SVA R package was used to remove batch effects using the combat algorithm (Leek et al., 2012). Two-dimensional principal component analysis (PCA) was used to evaluate whether the batch effect had been removed. Outlier samples were detected and removed by hierarchical cluster analysis with average linkage. Limma R package was used to investigate the DEGs in the training dataset, and genes with a false discovery rate <0.05 and absolute log2 fold change >0.5 were considered DEGs.

### Construction of Co-Expression network

We used DEGs to construct a gene co-expression network *via* WGCNA R package (Langfelder and Horvath, 2008). We constructed a scale-free network (*R* ^2^ = 0.85) based on the following criteria: the soft-thresholding power *β* was set as 20, the minimum number of genes in the modules was 30, and the threshold for cut height to merge possible similar modules was 0.25. To further analyze the module, we calculated the module eigengene, which represents the expression of all genes in a given module. The correlation between the module eigengene and the genes was defined as the module membership, and the correlation between the genes and clinical traits was defined as the gene significance. Model eigengene values were correlated with control and IPF groups by Pearson’s correlation. Finally, the module most highly associated with IPF groups was selected for further analysis.

### Functional enrichment analysis

For further understanding the function of the genes in the most related module for IPF, we conducted Gene Ontology (GO) and Kyoto Encyclopedia of Genes and Genomes (KEGG) enrichment analyses using Clusterprofiler R package (Wu et al., 2021). The ontology of the GO analysis contains three categories: molecular function, biological process, and cellular component. Adjusted *p* < 0.05 was set as the cutoff to identify significantly enriched pathways.

### Screening of candidate diagnostic biomarker

To screen candidate diagnostic biomarkers, we identified genes with high within-module connectivity of the modules (|module membership| > 0.8) and |gene significance| > 0.6 as hub genes and used them to build the least absolute shrinkage and selection operator (LASSO) and support vector machine-recursive feature elimination (SVM-RFE) algorithms (Huang et al., 2014). The LASSO algorithm, with penalty parameter tuning conducted by 10-fold cross-validation, was applied with the glmnet R package. The SVM-RFE algorithm was established by e1071 R package to search for lambda with the smallest classification error to select appropriate features, and the k-fold cross-validation was set as 10. The overlapping genes between the two algorithms were selected as candidate diagnostic biomarkers, and their expression was further validated by the validation datasets (GSE110147, GSE32537).

## Verification of diagnostic markers

The area under the curve and the receiver operating characteristic (ROC) curve were plotted with the pROC package to evaluate the capability of candidate diagnostic biomarkers between controls and IPF. We randomly divided samples from the training dataset into a training set and a test set (7:3) using the createDataPartition function in the caret R package and constructed the diagnostic model using the logistic regression algorithm in the training set. We used the test set and validation datasets to evaluate the capability of the diagnostic model between IPF and controls *via* ROC analysis.

## Results

### Removal of batch effects and identification of DEGs

**FIGURE 2:**
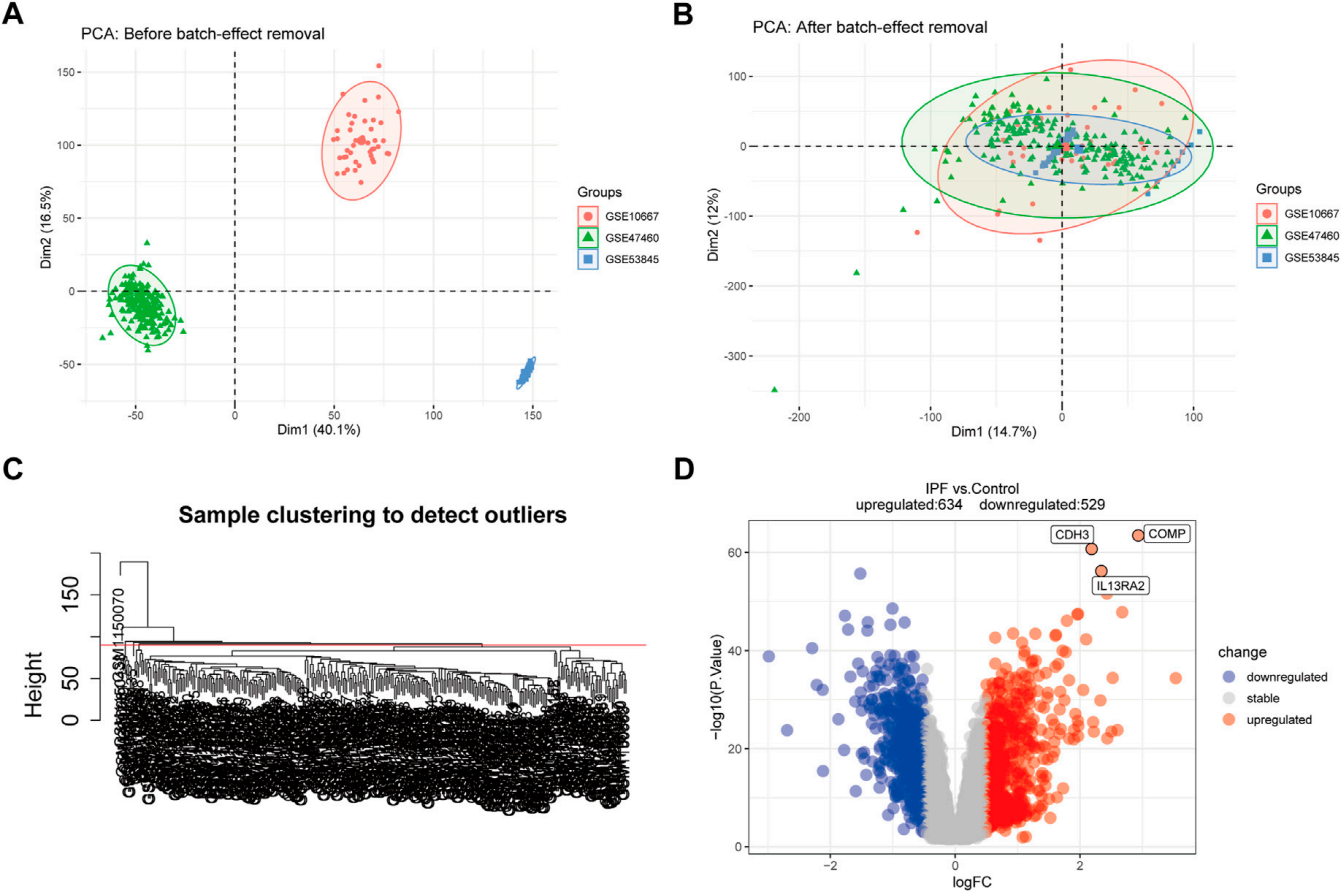
Principal component analysis (PCA) and differential expression analysis (DEGs). **(A,B)** Two-dimensional PCA cluster diagram before and after removing the batch effect. **(C)** Sample clustering to detect outliers. **(D)** The volcano plot of DEGs in training dataset, and the interested genes were plot in the volcano plot. Red represents upregulated DEGs, blue represents downregulated DEGs, and grey represents no significant difference genes.

The training dataset sample distributions before and after the removal of batch effects were visualized in a two-dimensional PCA cluster diagram (Figures 2A,B), and the scatter plot of PCA based on normalized expression showed that batch processing effects were eliminated (Figure 2B). 15 outlier samples were removed by hierarchical cluster analysis (cutHeight = 90; Figure 2C), and a total of 1,163 DEGs—529 downregulated and 634 upregulated genes—were identified in the training dataset (Figure 2D and Supplementary Table 1).

### Construction of the weighted Co-expression network and identification of key modules

**FIGURE 3:**
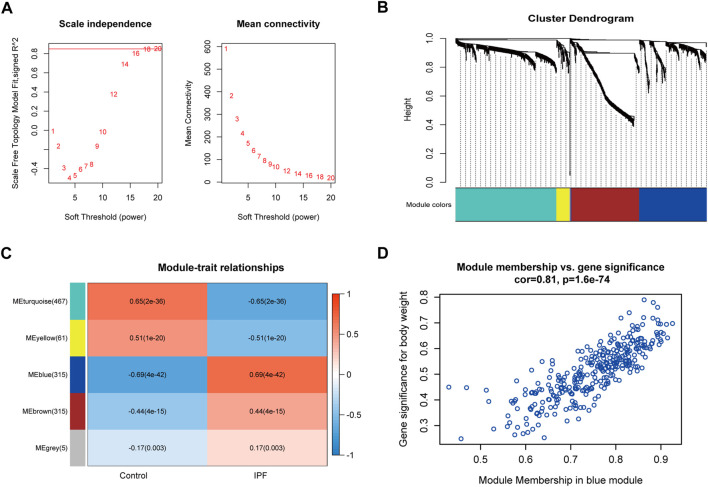
Co-expression modules construction and key module identification. **(A)** Determination of soft-thresholding power in the WGCNA. Analysis of the scale-free fit index for various soft-thresholding powers (*β*) and analysis of the mean connectivity for different soft-thresholding powers. **(B)** Clustering dendrogram of genes based on a dissimilarity measure (1-TOM). **(C)** Heatmap of the correlation between module eigengenes and clinical traits (each cell contained the correlation coefficient and corresponding *p*-value). **(D)** Scatter plot for correlation between the module membership and gene significance of blue module (one dot represents one gene).

A total of 292 samples and 1,163 DEGs were used to construct the co-expression network analysis. The power of *β* = 20 (scale-free *R* ^2^ = 0.85) was selected as the correlation coefficient threshold to identify the module-trait relationship (Figure 3A). A total of five modules were identified (Figure 3B). The blue module, which included 315 genes, had the strongest association with IPF (Figure 3C), and 59 genes with high connectivity in the blue module were selected for further analysis based on the cutoff criteria (|gene significance| > 0.6 and |module membership| > 0.8; Figure 3D and Supplementary Table 2).

### GO and KEGG pathway analyses

**FIGURE 4:**
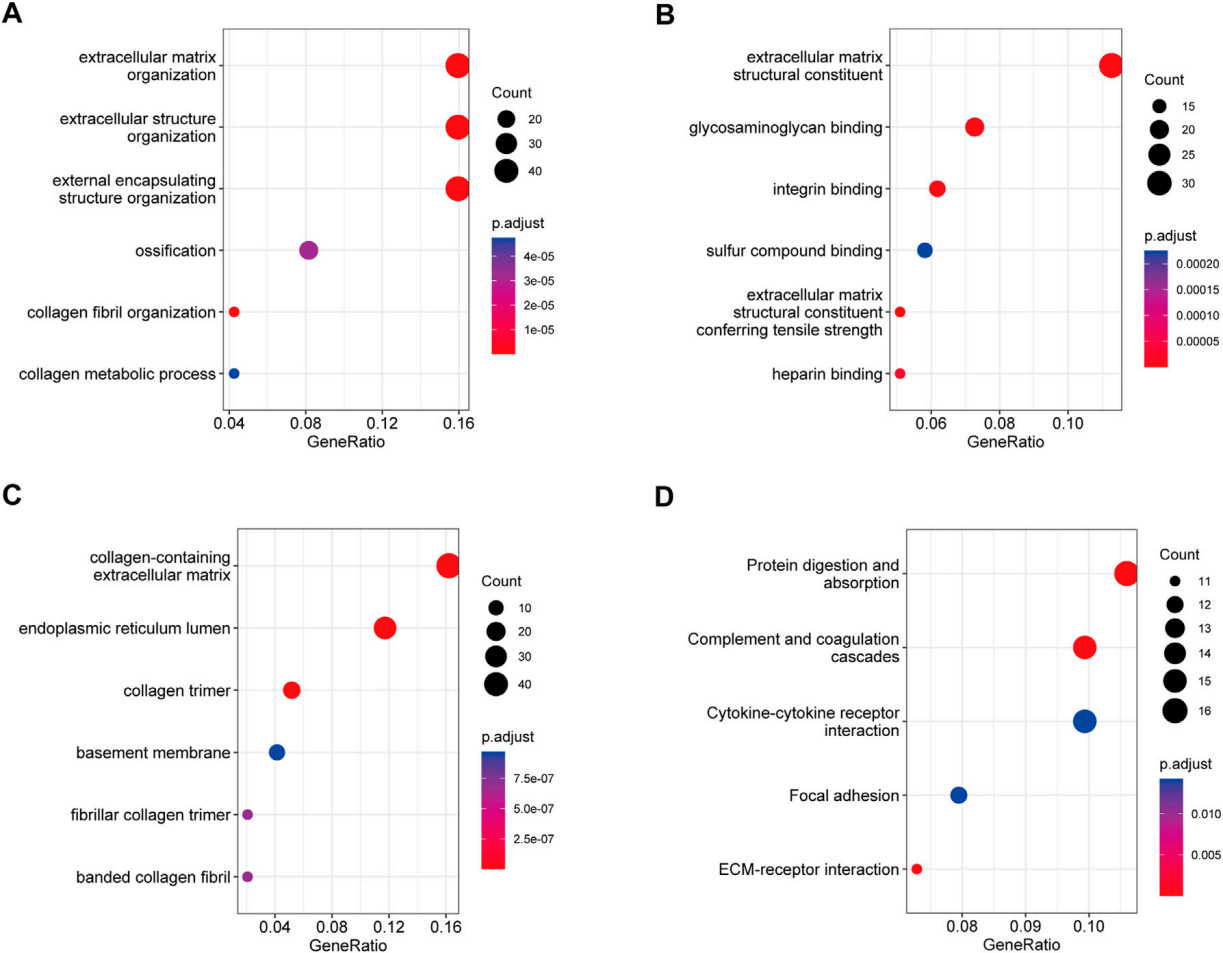
GO and KEGG pathway analysis of blue module genes. **(A)** Biological process analysis. **(B)** Molecular function analysis. **(C)** Cellular component analysis. **(D)** KEGG pathway analysis.

In the biological process category (Figure 4A), the genes were mainly enriched in ECM organization, extracellular structure organization, and external encapsulating structure organization. In the molecular function category (Figure 4B), the genes were mainly enriched in ECM structural constituent, endopeptidase activity, and glycosaminoglycan binding. In the cellular component category (Figure 4C), the genes were mainly enriched in collagen-containing ECM, endoplasmic reticulum lumen, and collagen trimer. KEGG pathway analysis revealed that protein digestion and absorption, complement and coagulation cascades, and ECM-receptor interaction were the most significant pathways (Figure 4D). These results indicate that blue module genes might impact the development of IPF by influencing these biological processes and signaling pathways.

### Screening and verification of diagnostic markers

**FIGURE 5:**
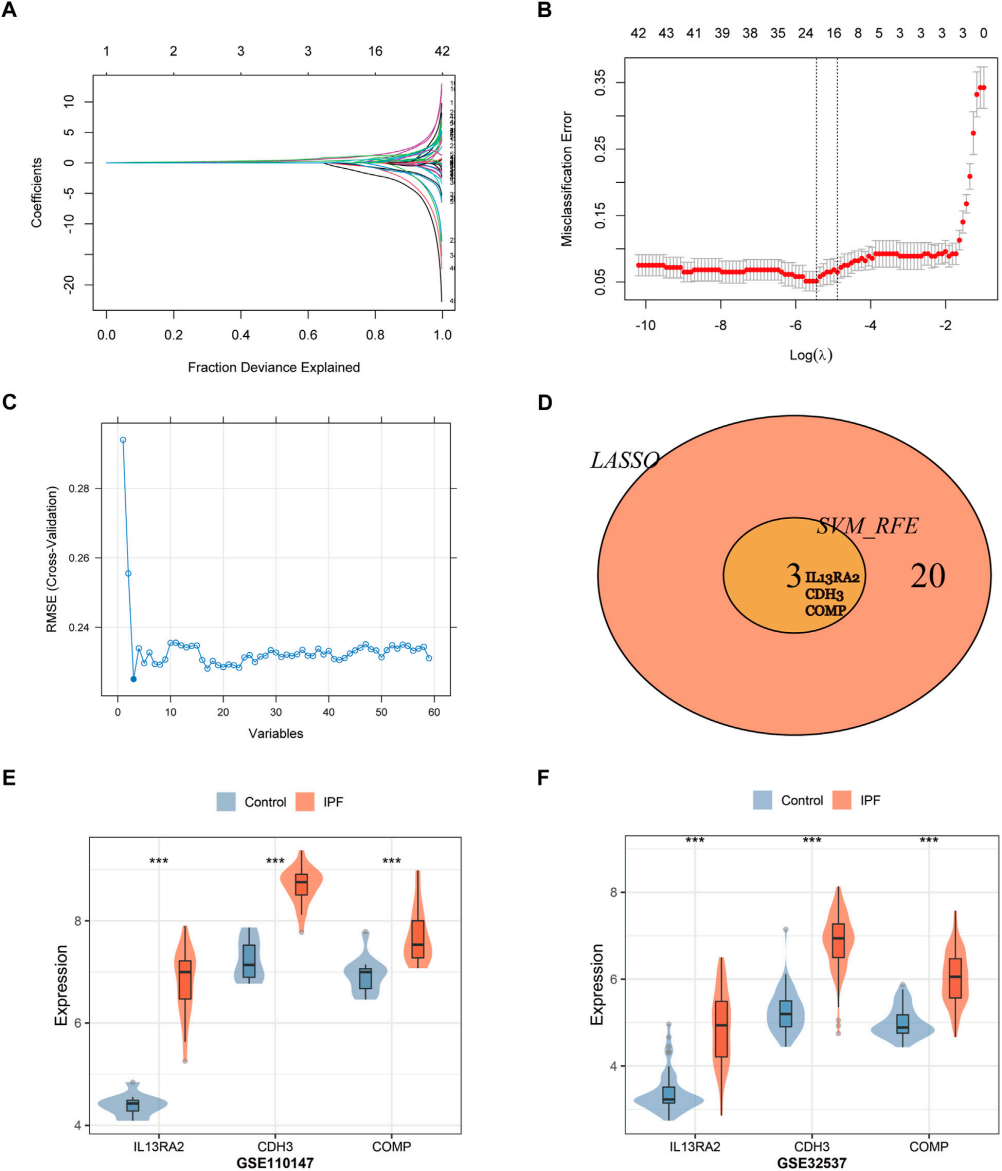
Two algorithms were used for gene signature selection. **(A)** LASSO algorithm to screen diagnostic markers. Different colors represent different genes. **(B)** The gene signature selection of optimal parameter (lambda) in LASSO algorithm. **(C)** The root mean square error of the estimate generation for the SVM-RFE algorithm. **(D)** Venn diagram shows the intersection of signature genes obtained from LASSO and SVM-RFE algorithms. **(E,F)** Validation of the expression levels of candidate diagnostic biomarkers in the validation datasets (GSE110147, GSE32537).***p < 0.001,*t*-test was used to evaluate the statistical significance of differences.

A total of 23 genes were identified as signature genes by the LASSO algorithm (Figures 5A,B), and three genes were identified by the SVM-RFE algorithm (Figure 5C). Interleukin-13 receptor alpha 2 (IL13RA2), cadherin 3 (CDH3), and Cartilage oligomeric matrix protein (COMP) were selected as signature genes by both the LASSO and SVM-RFE algorithms (Figure 5D), and they were identified as candidate diagnostic markers. The expression of the three genes was tested in the validation datasets, and it was notably higher in IPF lung tissue (*p* < 0.001; Figures 5E,F).

### Construction and verification of the IPF diagnostic model

**FIGURE 6:**
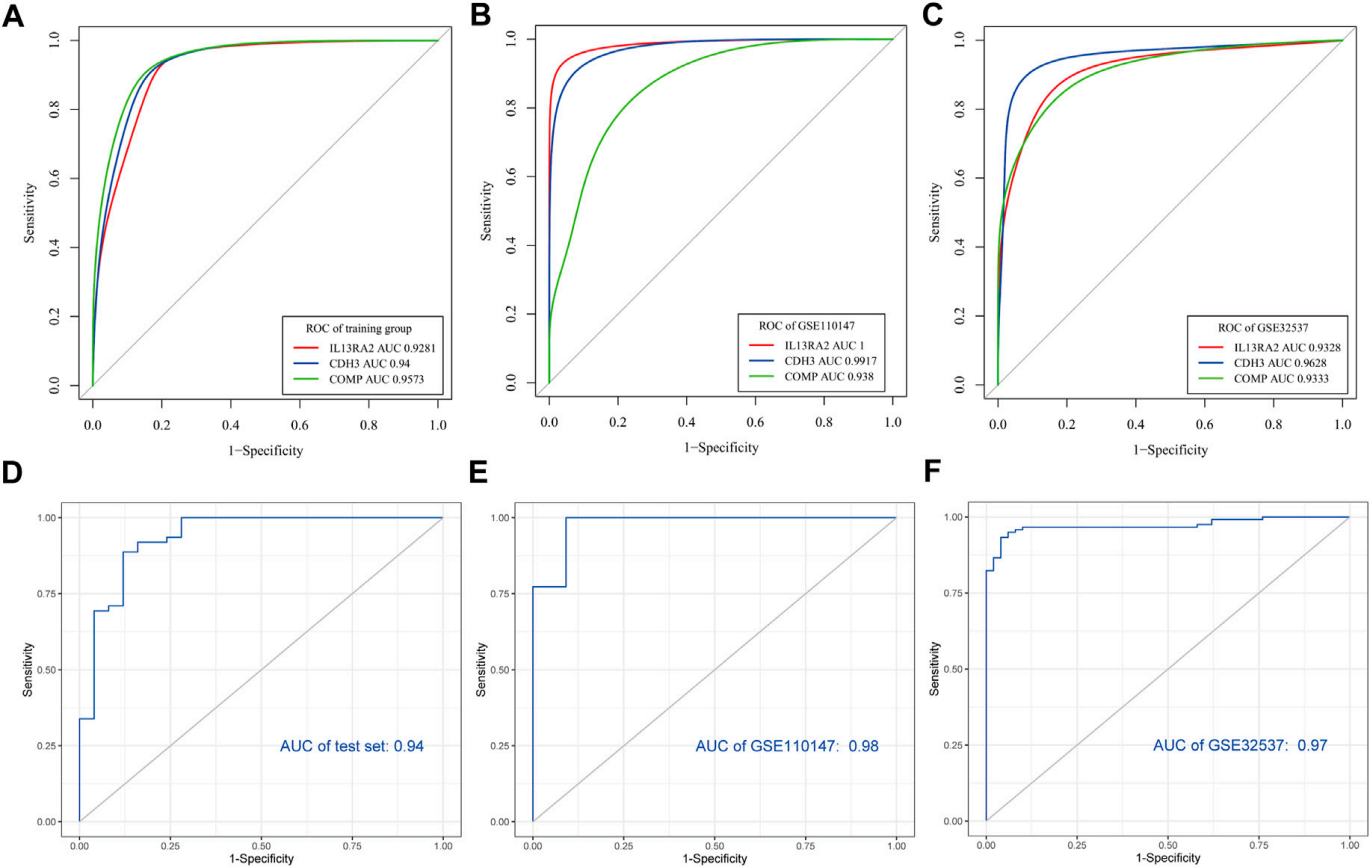
Verification of potential diagnostic signature of IPF. Verification of potential diagnostic signature of IPF. **(A–C)** The ROC curve of the diagnostic efficacy verification of diagnostic markers in the training dataset and validation datasets (GSE110147, GSE32537). **(D–F)** The ROC curves of the diagnostic markers in the test set and validation datasets (GSE110147, GSE32537).

Every area under the ROC curve (AUC) of IL13RA2, CDH3, and COMP was greater than 0.9 in training and validation datasets (GSE110147) (Figures 6A–C). When the three genes were combined into one variable, the area under the curve in the test set was 0.94 (Figure 6D). It is important to note that we also used external test datasets to validate the diagnostic value of the three-gene signature. The area under the curve for the three-gene signature was 0.98 in GSE110147 (Figure 6E) and 0.97 in GSE32537 (Figure 6F), which indicated that the three-gene signature had high diagnostic value.

## Discussion

Numerous studies have found that dysregulated genes are involved in the progression of IPF and might be potential diagnostic biomarkers of IPF (Ley et al., 2014; Martinez et al., 2017). However, most of these studies have focused on the diagnostic value of a single biomarker, and small sample sizes may limit the clinical application of these biomarkers. Integrating multiple biomarkers and increasing sample sizes with developing high-throughput technologies and bioinformatics may improve predictive accuracy. In this study, we constructed a novel gene signature for diagnosing IPF by integrating multiple datasets and combining multiple statistical methods. Three genes (IL13RA2, CDH3, and COMP) were identified as independent diagnostic biomarkers. An integrated three-gene signature was built based on their regression coefficients and expression profiles, ROC analysis indicated that the signature had high diagnostic accuracy, as confirmed by two external test datasets.

The mouse bleomycin-induced fibrosis model is generally viewed as the standard in modeling pulmonary fibrosis (Jenkins et al., 2017). Recent studies revealed that genes related to the mouse bleomycin-induced fibrosis model and human IPF have much in common (Bauer et al., 2015; Toren et al., 2021). Interleukin (IL)-13 is a type 2 cytokine with important roles in inflammatory and fibrotic diseases (Gieseck et al., 2018; Bieber, 2020). IL-13 receptor α2 (IL-13Rα2), which has great affinity for IL-13, acts as a non-signaling decoy receptor (Chandriani et al., 2014; Ulzii et al., 2019). IL-13 and its receptors are increased in the blood and lung tissue of IPF patients (Murray et al., 2008; Chandriani et al., 2014). TGF-β is upregulated and activated in fibrotic diseases and is considered a central mediator of fibrogenesis (Györfi et al., 2018; Frangogiannis, 2020). Previous studies have found that IL-13 signals through IL-13R2 to induce transforming growth factor beta (TGF-β) and fibrosis progression (Fichtner-Feigl et al., 2008), and silencing of IL-13Rα2 reduced TGF-β production and lung fibrosis (Fichtner-Feigl et al., 2006). In contrast, Robert V et al. found that overexpression of IL-13Rα2 inhibited the IL-13 induction of fibrotic markers and bleomycin-induced pulmonary fibrosis (Lumsden et al., 2015). IL-13Rα2 deficiency had been shown to lead to increases in collagen deposition (Wilson et al., 2007). Given these contrasting possibilities, it is vital to interpret the meaning of dysregulated IL-13R2 expression in IPF and the function of IL-13Rα2. The expression of IL13RA2 was significantly higher in IPF and acute exacerbation of IPF (AE-IPF) compared with control samples, but there was no significant difference between IPF and AE-IPF (Konishi et al., 2009). These results indicate that IL13RA2 may serve as a signature for IPF, but can’t predict the onset of AE-IPF.

CDH3, also known as placental cadherin, is a classic cell-to-cell adhesion molecule that regulates multiple cellular homeostatic processes in normal tissue (Vieira and Paredes, 2015). High CDH3 expression is associated with tumor progression in invasive epithelial tumors (Ribeiro et al., 2010) and non-small-cell lung cancer (Imai et al., 2018). A well-known mechanism associated with CDH3-induced cancer cell invasion is MMP activation (Ribeiro et al., 2010; Imai et al., 2018). MMPs have been implicated in the pathogenesis of IPF, and most MMPs promote the development of IPF (Craig et al., 2015; Mahalanobish et al., 2020). CDH3 was expressed in aberrant basaloid cells in the IPF lung (Adams et al., 2020; Travaglini et al., 2020) and overexpressed in all three stages of IPF (early-, progressive, and end-stage IPF) (Ghandikota et al., 2022), but was not found to be differentially expressed in bleomycin-treated animals (Bauer et al., 2015). It was not further investigated due to lack of translational relevance.

COMP is an ECM glycoprotein that plays a role in fibrillogenesis and collagen secretion (Rockey et al., 2015; Posey et al., 2018). Dysregulated COMP expression is involved in numerous diseases (Posey et al., 2018), including fibrosis (Agarwal et al., 2013; Vuga et al., 2013). COMP expression was increased in skin fibroblasts and lung epithelial cell induction by TGF-β, silencing COMP expression in human lung fibroblasts was associated with a reduction in TGF- β1 (Agarwal et al., 2013; Vuga et al., 2013). Once fibrosis is initiated, COMP and TGF-β constitute a mutual positive regulation loop (Agarwal et al., 2013; Cui and Zhang, 2022). Serum COMP was upregulated in IPF patients and correlated with declines in force vital capacity, indicating that it is a potential biomarker for disease activity (Vuga et al., 2013). K. Miller, et al. found that the expression of COMP was significantly increased in bleomycin-treated mice, while tissue elastance and lung function testing showed significant differences compared to control group. However, there was no significant difference between bleomycin treated WT and COMP-KO mice. It indicated COMP maybe a biomarker for pulmonary fibrosis but not a causative factor in bleomycin-induced fibrosis (Miller et al., 2019).

IPF is a chronic, progressive lung disease characterized by the progressive deposition of ECM proteins. Available evidence suggests that the ECM plays a central role in the pathogenesis of IPF (Hewlett et al., 2018). WGCNA is a bioinformatics application for exploring the relationships between clinical traits and co-expression modules (Langfelder and Horvath, 2008). In the present study, one module mostly associated with IPF was found by WGCNA. Functional enrichment analysis revealed that the module’s genes involved in multiple biological processes, including ECM organization, extracellular structure organization, and collagen metabolism by protein digestion and absorption signaling pathways. These results suggest that dysregulated expression of these genes could play important roles in orchestrating the development of IPF by influencing ECM-associated pathways.

Although the three-gene signature constructed here appears to be a potential diagnostic signature for IPF, this study has some limitations. First, the expression and exact mechanisms of the biomarkers should be further investigated experimentally. Second, the diagnostic value of the three-gene signature was only tested in microarray datasets in this study and should be further validated with clinical data. Third, other genes in the clinically significant module were not studied in this study.

In conclusion, we revealed the involvement of the key gene co-expression module and functional pathways in the pathogenesis of IPF. In addition, we identified IL13RA2, CDH3, and COMP as potential biomarkers of IPF and constructed an integrated three-gene signature of IPF. This work provides further insights into the underlying molecular mechanisms and diagnosis of IPF.

## Data Availability

The datasets presented in this study can be found in online repositories. The names of the repository/repositories and accession number(s) can be found in the article/Supplementary Material.

## Abbreviations

AE-IPF, acute exacerbation of IPF; AUC, area under the receiver operating characteristic curve; COMP, Cartilage oligomeric matrix protein; CDH3, cadherin three; DEGs, differentially expressed genes; ECM, extracellular matrix; GO, Gene Ontology; KEGG, Kyoto Encyclopedia of Genes and Genomes; PCA, principal component analysis; IPF, idiopathic pulmonary fibrosis; WGCNA, weighted correlation network analysis; LASSO, least absolute shrinkage and selection operator; SVM-RFE, support vector machine-recursive feature elimination; IL13RA2, interleukin-13 receptor alpha two; MMP, matrix metalloproteinase; TGF-β, transforming growth factor beta; ROC, receiver operating characteristic.
