# Supplementary material for: A potential three-gene-based diagnostic signature for idiopathic pulmonary fibrosis
Source: Front Genet. 2023 Jan 6;13:985217. doi: 10.3389/fgene.2022.985217 (PMC9857386; doi:10.3389/fgene.2022.985217)
Supplement: Supplementary file 1 [file Table1.docx]

| **GEO accession** | **Publication year** | **Sample size**  **(IPF/Control)** | **Source** | **Platform** |
| --- | --- | --- | --- | --- |
| [GSE110147](https://www.ncbi.nlm.nih.gov/geo/query/acc.cgi?acc=GSE110147) | 2018 | 22/11 | Lung tissue | Affymetrix Human Gene 1.0 ST Array |
| [GSE53845](https://www.ncbi.nlm.nih.gov/geo/query/acc.cgi?acc=GSE53845) | 2014 | 40/8 | Lung tissue | Agilent-014850 Whole Human Genome Microarray 4x44K G4112F |
| [GSE47460](https://www.ncbi.nlm.nih.gov/geo/query/acc.cgi?acc=GSE47460) | 2013 | 122/91 | Lung tissue | Agilent-028004 SurePrint G3 Human GE 8x60K Microarray |
| [GSE32537](https://www.ncbi.nlm.nih.gov/geo/query/acc.cgi?acc=GSE32537) | 2013 | 119/50 | Lung tissue | Affymetrix Human Gene 1.0 ST Array |
| [GSE10667](https://www.ncbi.nlm.nih.gov/geo/query/acc.cgi?acc=GSE10667) | 2009 | 31/15 | Lung tissue | Agilent-014850 Whole Human Genome Microarray 4x44K G4112F |
